# Cytokines induced memory-like NK cells engineered to express CD19 CAR exhibit enhanced responses against B cell malignancies

**DOI:** 10.3389/fimmu.2023.1130442

**Published:** 2023-05-03

**Authors:** Bailin He, Qiusui Mai, Yunyi Pang, Shikai Deng, Yi He, Rongtao Xue, Na Xu, Hongsheng Zhou, Xiaoli Liu, Li Xuan, Chengyao Li, Qifa Liu

**Affiliations:** ^1^Department of Hematology, Nanfang Hospital, Southern Medical University, Guangzhou, China; ^2^Department of Transfusion Medicine, School of Laboratory Medicine and Biotechnology, Southern Medical University, Guangzhou, China; ^3^Department of Rheumatology and Immunology, The Third Affiliated Hospital, Southern Medical University, Guangzhou, China

**Keywords:** natural killer (NK) cells, memory-like NK cells, cellular immunotherapy, B cell malignancies, chimeric antigen receptor (CAR)

## Abstract

CD19 chimeric antigen receptor (CAR) engineered NK cells have been used for treating patients with relapsed and/or refractory B cell malignancies and show encouraging outcomes and safety profile. However, the poor persistence of NK cells remains a major challenge for CAR NK cell therapy. Memory-like NK cells (MLNK) induced by IL-12, IL-15, and IL-18 have shown enhanced and prolonged responses to tumor re-stimulation, making them an attractive candidate for adoptive cellular immunotherapy. Here, we show efficient and stable gene delivery of CD19 CAR to memory-like NK cells using retroviral vectors with transduction efficiency comparable to those achieved with conventional NK cells. Analysis of surface molecules revealed a distinct phenotypic profile in CAR engineered memory-like NK cells (CAR MLNK), as evidenced by increased expression of CD94 and downregulation of NKp30 as well as KIR2DL1. Compared to conventional CAR NK cells, CAR MLNK cells exhibited significantly increased IFN-γ production and degranulation in response to CD19^+^ target cells, resulting in enhanced cytotoxic activity against CD19^+^ leukemia cells and lymphoma cells. Furthermore, memory properties induced by IL-12/-15/-18 improved the *in vivo* persistence of CAR MLNK cells and significantly suppressed tumor growth in a exnograft mouse model of lymphoma, leading to prolonged survival of CD19^+^ tumor-bearing mouse. Altogether, our data indicate that CD19 CAR engineered memory-like NK cells exhibited superior persistence and antitumor activity against CD19^+^ tumors, which might be an attractive approach for treating patient with relapse or refractory B cell malignancies.

## Introduction

Chimeric antigen receptor (CAR) has been used to redirect the specificity of NK cells against various cancers, including hematologic malignancies and solid tumors (1–4). CD19-specific CAR engineered NK cells have been reported to induce complete remission in 63% (7/11) of patients with relapsed or refractory B cell malignancies, including chronic lymphocytic leukemia and non-Hodgkin’s lymphoma (5). Moreover, allogeneic CAR NK cells do not produce graft-versus-host disease (GVHD) and have not been associated with serious cytokine release syndrome (CRS) and immune effector cell-associated neurotoxicity syndrome (ICANS), which are common side effects of CAR T cell therapy (5–7). However, the poor post-infusion persistence of NK cells remains a major challenge for CAR NK cell therapy. Although IL-2 has been used for expanding adoptive NK or T cells in patients, systemic IL-2 administration can cause severe toxicities like vascular leak syndrome, and lead to the activation of regulatory T cells which might impair the function of NK cells (8–10). Thus, developing strategies to improve the maintenance of NK cells is particularly important for enhancing the therapeutic efficacy of CAR NK cell therapy.

CAR T cells with a long-lived memory phenotype, like stem cell memory T cells (T_SCM_) and central memory T cells (T_CM_), are correlated with durable remissions in patients with hematological malignancies (11–13). However, memory NK cells have not been fully identified or characterized. Peripheral blood NK cells can be briefly activated with IL-12, IL-15 and IL-18 to induce differentiation into memory-like NK cells, associated with enhanced responses when simulated through tumor targets for weeks to months after pre-activation (14, 15). IL-12/-15/18 cytokines induced memory-like NK cells were reported as safe and able to induce complete remissions in patients with relapse and/or refractory acute myeloid leukemia (AML). The adoptive transferred memory-like NK cells (MLNKs) were able to expand and persist in the blood and bone marrow of patients for at least 21 days after infusion (16, 17). Given that MLNKs are long-lived and have relatively higher antitumor activity compared with conventional NK cells (18–20), we thus investigated whether combining memory-like NK cells with CD19-specific CAR could exhibit enhanced and prolonged antitumor activity against CD19^+^ B cell malignancies.

In this study, we demonstrated that peripheral blood NK cells pre-activated with IL-12/-15/-18 prior to transduction with CD19-specific CAR resulted in a more activated, non-exhausted, and memory-like phenotype associated with enhanced response to CD19-positive targets. CD19 CAR engineered MLNK cells exhibited significantly increased persistence and superior antitumor efficacy against CD19^+^ tumor cells compared with conventional CAR NK cells *in vitro* and *in vivo*. Thus, combining CD19 CAR with cytokine induced memory-like NK cells might be a promising strategy for treating patients with relapse or refractory CD19^+^ B cell malignancies.

## Materials and methods

### Cells and culture

CD19^+^ Nalm6 (B cell precursor leukemia cell line), CD19^+^ Raji (human Burkitt’s lymphoma cell line) and CD19^-^ K562 (human myelogenous leukemia cell line) were obtained from the American Type Culture Collection. To establish the K562-FFLuc, Nalm6-FFLuc and Raji-FFLuc cells, K562, Nalm6 and Raji cells were transduced with the lentiviral supernatant encoding Firefly Luciferase and GFP. To establish engineered K562 feeder cells co-expressing CD48, CD137L and membrane bound IL-21 (mbIL-21), K562 cells were serially transduced with retroviral supernatants harboring mbIL-21, CD48 and CD137L transgenes. Tumor cells were cultured in RPMI 1640 medium supplemented with 10% heat-inactivated fetal bovine serum (FBS) (Gibco), 100 units/mL penicillin, and 100µg/mL streptomycin (Invitrogen) at 37°C in a humidified 5% CO2 incubator.

### Cytokines and antibodies

Recombinant human IL-12 and IL-15 (PeproTech) and IL-18 (R&D Systems) was used to stimulate NK cells at the beginning of the culture. Alexa Fluor 700 anti-human CD3 antibody (clone OKT3), BV421 anti-human CD56 antibody (clone NACM), PC/Cyanine7 anti-human CD16 antibody (clone 3G8), PE/Cyanine7 anti-human CD25 antibody (clone BC96), PerCP anti-human CD69 antibody (clone FN50), APC anti-human CD158 (KIR2DL1) antibody (cloneHP-MA4), APC anti-human NKp30 antibody (clone 730-15), APC anti-human CD335 (NKp46) antibody (clone 9E2), APC anti-human CD94 antibody (clone DX22), PE anti-human CD314 (NKG2D) antibody (clone 1D11), Alexa Fluor 488 anti-human CD279 (PD-1) antibody (clone EH12.2H7), Brilliant Violet 421 anti-human CD36/Tim-3 antibody (clone F38-2E2LAG3), PE/Cyanine5 anti-human NKG2A/CD159a antibody (clone S19004C), PE anti-human NKG2C/CD159c antibody (clone S19005E), APC anti-Human IFN-γ antibody (Clone B27) and PE/Cyanine7 anti-human CD107a (LAMP-1) antibody (clone H4A3) were purchased from Biolegend. PE Goat anti-human IgG(H+L) F(ab’)2 fragment antibody was purchased from Jackson ImmunoResearch.

### Retrovirus production and anti-CD19 CAR NK cell generation

To produce CD19 CAR retrovirus, 293T cells were transfected with pSFG retroviral vector plasmid encoding 3^rd^ generation CD19-specific CAR containing CD28 and 4-1BB costimulation domain, PegPam3 plasmid, and RDF plasmid, as previously described (21). NK cells were isolated from healthy donor derived PBMC using NK Cell Isolation Kit (Milteyi Biotec). Purified NK cells were cultured with 100Gy-irradiated K562 feeder cells in SCGM Medium (CellGenix) with 10% FBS, 2 mM L-glutamine, 10 ng/mL IL-15. Activated NK cells were harvested on day 4 and transduced with CD19 CAR retrovirus in plates coated with RetroNectin (TaKaRa). Medium containing 10 ng/mL of IL-15 were changed every 3-4 days till day 14 of cultivation. Total cell numbers were counted over time using trypan blue (n=4). To determine the percentage of NK cells and expression of CAR, cells were stained with CD3, CD56, CD16, and anti-human IgG(H+L) F(ab’)2 fragment and analyzed by flow cytometry on day 3 and day 10 post transduction (n=4).

### Transduction of MLNK cells with CD19 CAR

**Figure 1:**
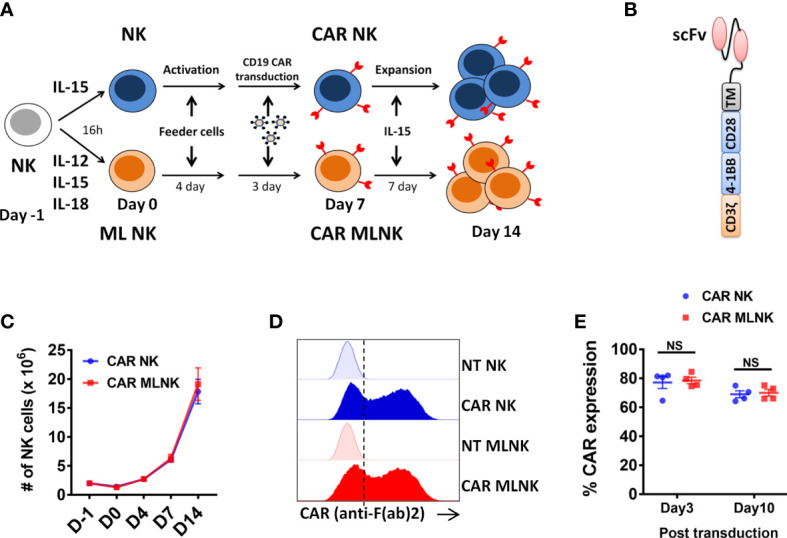
Generation of CD19 CAR engineered memory-like NK cells. **(A)** Schematic diagram of generation of CAR MLNK cells and conventional CAR NK cells. NK cells were pre-activated with IL-12, IL-15 and IL-18 or were control treated (IL-15 only) on day -1, stimulated with engineered K562 feeder cells on day 0, and transduced with retroviral vectors on day 4. NK cells were cultured for a total of 14 days to allow for expansion. **(B)** Schematic representation of CD19 CAR construct containing the CD19 single chain variable fragment (scFv), CD28 transmembrane domain, CD28 and 4-1BB costimulatory domains and signaling domain CD3zeta. **(C)** Absolute number of CAR NK cells or CAR MLNK cells shown over time (n=4). **(D)** Representative FACS histogram plots were shown for CAR expression on CAR NK cells, CAR MLNK cells, NT NK cells and NT MLNK cells on day 3 post transduction. **(D)** Summarized histogram showing the transduction efficiency of CAR on day 3 and day 10 post transduction (n=4). ns, no significance.

To generate cytokine induced MLNK cells, peripheral blood NK cells from 4 healthy donors were isolated and pre-activated with 10 ng/mL IL-12, 10 ng/mL IL-15, and 50 ng/mL IL-18 for 16-18 hours, as previously described (15–17, 22), and conventional NK cells were pre-activated with 10 ng/mL IL-15 only (**Figure 1A**). After pre-activation, NK cells or MLNK cells were washed twice and cocultured with irradiated K562 feeder cells and 10 ng/mL of IL-15 for 4 days. The expanded MLNK and conventional NK cells were then transduced with CD19 CAR retrovirus as above. All CAR transduced and non-transduced MLNK (CAR MLNK and NT MLNK) and conventional NK (CAR NK and NT NK) cells were maintained in complete media with IL-15 every 3 to 4 days, and the phenotype profile and function of NK cells were explored on day 10 post-transduction if not indicated (n=4).

### Degranulation assay

To assess the degranulation of NK cells against CD19^+^ target cells Raji, CAR NK/MLNK or NT NK/MLNK cells (total NK 5 × 10^5^) were cocultured with Raji cells (1 × 10^5^) in the presence of anti-human CD107a (n=4). After 1 hour, brefeldin A and monensin (Biolegend) were added and incubated for an additional 4 hours. Cells were then washed and stained with CD3, CD56, CD16 on ice for 30 min. The cells were washed with FACS buffer and analyzed using flow cytometry.

### Intracellular cytokine staining

For cytokine release analysis, CAR NK/MLNK and NT NK/MLNK cells were stimulated with Raji cells for 4 hours in the presence of monensin and brefeldin A (n=4). For the unstimulated control, NK cells were treated with the secretion inhibitors, but not tumor cells. After the incubation, cells were harvested and stained with CD3, CD16, CD56 antibodies and anti-human IgG(H+L) F(ab’)2 fragment for 30 minutes. After cell fixation and permeabilization with the BD Fixation/Permeabilization Kit, cells were stained with IFN-γ for 30 minutes on ice in the dark. After washing once with 1 ml of FACS buffer, cells were resuspended in 300 µl FACS buffer for acquisition.

### Luciferase-based cytotoxicity assays

To assess the cytotoxic capacity of NK cells, firefly luciferase reporter was used to be introduced into target cells (CD19^+^ Raji, CD19^+^ Nalm6 and CD19^-^ K562) and detected with substrate D-luciferin for measuring changes. NK cells (CAR NK/MLNK and NT NK/MLNK cells) on day 10 after transduction were harvested and cocultured with Raji, Nalm6 or K562 expressing firefly luciferase at 5:1, 2:1, 1:1, 0.5:1 of Effector to Target (E:T) ratios for 6 hours (n=4). Luminescence intensity was measured after addition of D-Luciferin substrate using luminescence microplate reader. The luminescence readings are expressed as Relative Light Unit (RLU). The % killing of tumor cells by NK cells was calculated according to the following equation: % Target Lysis  = 100% × (RLU of untreated tumor cells – RLU of tumor cells cultured with NK cells)/RLU of untreated tumor cells.

### *In vitro* co-culture assay

CAR MLNK cells or conventional CAR NK cells harvested on day 10 after transduction were cocultured with CD19^+^ Raji expressing GFP for 3 days at the E:T=1:4 in the presence of 10 ng/mL IL-15 or not. Percentage of CAR NK/MLNK cells (GFP^-^ CD56^+^ CAR^+^) after coculture were analyzed using flow cytometry. Absolute number of CAR NK/MLNK cells in the co-culture were calculated by CountBright absolute counting beads (Thermo Fisher Scientific) *via* flow cytometry (n = 4).

### Animal experiment

All procedures with NOD-SCID-γ (NSG) mice were performed in accordance with the relevant institutional animal care and used Committee requirements. Animal experiments were approved by the Ethics Committee of the University of Southern Medical University. NSG mice were injected intravenously (IV) with 1 × 10^5^ Raji cells on day 0. On day 3, mice received IV injection of 5 × 10^6^ total NK cells in 200 uL PBS, containing 45% to 55% CAR^+^ NK cells (n = 10) or CAR^+^ MLNK cells (n = 11), or 200μL PBS (n = 9). To maintain NK cell survival in NSG mice that lacked homeostatic survival signals, IL-15 (0.5 ug per mice) was intraperitoneally injected every 3 days. On day 7, mice were anesthetized, peripheral blood was collected and assessed for the presence of NK cells and GFP^+^ Raji cells, and the serum was used for IFN-γ and IL-6 measurement by ELISA according to the instruction. Tumor burden were monitored *in vivo* over time by quantitation of photon emission using Xenogen *In vivo* Imaging System (IVIS). Body weight of mice was measured every 2 days.

### Statistical analysis

Statistical analysis was performed using GraphPad Prism v7. *P* values were calculated using the Student *t*-test for comparison between two groups or the one-way ANOVA test with Dunnett’s multiple comparisons for comparison more than two groups. Kaplan-Meier survival curves of animal experiments were analyzed using log-rank. *P* values < 0.05 were considered as statistically significant. Where not otherwise indicated, results are presented as mean ± standard error of the mean (SEM). Significance of findings was defined as: ns, no significance; * *P* < 0.05; ** *P* < 0.01; *** *P* < 0.001.

## Results

### Generation of CD19 CAR engineered memory-like NK cells

Purified NK cells from peripheral blood were pre-activated with IL-12, IL-15 and IL-18 to generate memory-like NK cells or IL-15 to maintain survival as conventional NK cells, stimulated with engineered K562 feeder cells (**Supplementary Figure 1**), transduced with retrovirus encoding CD19-specific CAR, and cultured for a total of 14 days to allow for expansion and CAR expression (**Figures 1A, B**). There was no significant difference in the NK cell expansion between CAR-transduced MLNK cells and conventional CAR NK cells (**Figure 1C**, n=4). The median transduction efficiency of CAR on MLNK cells was up to 78.32%, which is similar to that of conventional NK cells on day 3 post transduction (77.17 ± 4.2%, **Figure 1D**, n=4). CAR expression on MLNK cells remained stable on day 10 post transduction (68.98 ± 4.31%, **Figure 1E**). Moreover, the NK purity and viability of CAR MLNK cells on day 14 of cultivation were more than 99% and 90%, respectively (**Supplementary Figure 2**). Taken together, we successfully generated CD19 CAR engineered memory-like NK cells with high transduction efficiency and viability.

### Immunophenotypic analysis of CAR engineered memory-like NK cells

**Figure 2:**
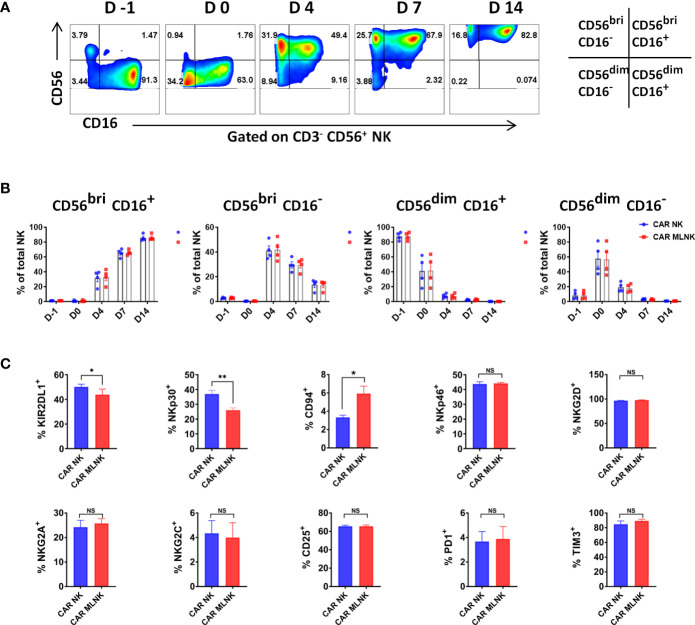
Phenotypic analysis of CAR engineered memory like NK cells. **(A)** Representative flow cytometry plots showing the distribution of NK cell subtypes in CAR modified memory-like NK cells based on the relative expression of CD56 and CD16 (n = 4). **(B)** Evolution of CD56^bright^ CD16^+^, CD56^bright^ CD16^-^, CD56^dim^ CD16^+^ and CD56^dim^ CD16^-^ NK cell subsets in CAR MLNK cells and CAR NK cells over time (n = 4). **(C)** Expression levels of KIR2DL1, CD94, NKp30, NKp46, NKG2A, NKG2D, NKG2C, CD25, PD1 and TIM3 on CAR MLNK cells and conventional CAR NK cells (n = 4). *P < 0.05; **P < 0.01; ns, no significance.

Next, the subtypes and functional biomarkers of CAR MLNK cells were analyzed. Four NK cell subsets including CD56^bright^ CD16^+^, CD56^bright^ CD16^-^, CD56^dim^ CD16^+^ and CD56^dim^ CD16^-^ cells were found during the generation and expansion of CAR MLNK cells (**Figure 2A**). The population of CD56^bright^ CD16^+^ cells was low before IL-12/-15/-18 activation, but constituted the majority population in CAR MLNK cells on day 14 (86.1 ± 4.1%, **Figure 2B**). In contrast, CD56^dim^ CD16^+^ NK subset, which is responsible for natural cytotoxicity and constitute the majority of NK cells in peripheral blood (87.8 ± 5.4%), were continuously decreased during *ex vivo* culture and declined to 0.08% on day 14. CD56^dim^ CD16^-^ cell fraction was markedly increased after IL-12/-15/-18 activation (57.0 ± 21.1%) but gradually declined till day 14 (0.69 ± 0.1%). The percentage of CD56^bright^ CD16^-^ immunoregulatory NK cells peaked on day 4 (41.8 ± 8.7%) and fall to 13.16% on day 14 of cultivation. A similar trend of the evolution of NK cell subsets was seen in conventional CAR NK cells and CAR MLNK cells (**Figure 2B**). To determine the phenotypic profiles of CAR MLNK cells, we used flow cytometry to characterize several important activating and inhibitory receptors (**Figure 2C**). The expression of natural cytotoxicity receptor NKp30 was decreased significantly in CAR MLNK cells compared to conventional CAR NK cells (26.0 ± 1.6% vs 36.9 ± 2.4%), but there is no appreciable difference of NKp46 between them (44.1 ± 0.6% vs 43.7 ± 1.5%). Lower levels of inhibitory receptor KIR2DL1 was also found on CAR MLNK cells. CD94, which triggers inhibition or stimulation signals when associated with NKG2A or NKG2C respectively, was up-regulated on CAR MLNK cells, but no significant change of NKG2A and NKG2C between CAR MLNK and CAR NK cells was found. In addition, expression of activation markers NKG2D, CD25 and CD69, as well as exhaustion markers PD1 and TIM3, on CAR MLNK cells and CAR NK cells were comparable. In summary, CAR modified memory like NK cells displayed a distinct phenotype profile with a higher expression of activating receptors NKp30 and NKp46 and lower levels of inhibitory receptor KIR2DL1.

### Preactivation with IL-12, IL-15 and IL-18 promoted CAR NK cell degranulation and IFN-γ production

**Figure 3:**
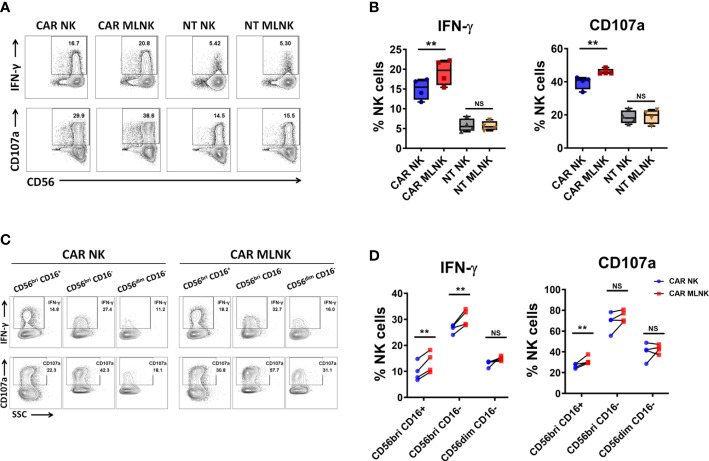
Functionality of CAR engineered memory-like NK cells. **(A)** CAR NK cells, CAR MLNK cells, NT NK cells or NT MLNK cells were cocultured with CD19^+^ Raji cells for 5h and harvested for analysis of the surface expression of CD107a and intracellular expression of IFN-γ. FACS plot showed one representative data from 4 healthy donors. **(B)** Summary data of the expression of CD107 and IFN-γ on CAR NK cells, CAR MLNK cells, NT NK or NT MLNK cells (n=4). **(C)** Representative FACS plots were shown for gating method for analysis of CD107 and IFN-γ on CD56^bri^ CD16^+^ NK cells, CD56^bri^ CD16^-^ NK cells or CD56^dim^ CD16^-^ NK cells after stimulated with Raji cells. **(D)** Summary data of the expression of CD107 and IFN-γ on CD56^bri^ CD16^+^, CD56^bri^ CD16^-^ or CD56^dim^ CD16^-^ CAR MLNK cells and CAR NK cells (n=4). **P < 0.01 ns, no significance.

To assessed whether IL-12/15/18 pre-activation could positively impact CAR NK-triggered responses, we evaluated the degree of IFN-γ intracellular expression and degranulation marker CD107a surface expression upon CD19^+^ Raji cell stimulation. The intrinsic anti-tumor activity of MLNKcells was observed in the background levels of IFN-γ and CD107a expression in NT MLNK cells (5.6 ± 0.6%, 19.1 ± 2.2%, respectively, n = 4), and their expression were more pronounced in CAR MLNK cells (19.3 ± 1.6%, 46.0 ± 0.9%, respectively), indicating that CAR transduction increase their antigen-specific antitumor activity (**Figures 3A, B**). Moreover, IFN-γ and CD107a expression were significant higher in CAR MLNK cells compared to conventional CAR NK cells stimulated with CD19^+^ Raji cells (19.3 ± 1.6% vs 15.0 ± 1.3%, 46.0 ± 0.9% vs 40.0 ± 2.0%, respectively, n = 4), while NT MLNK cells and NT NK cells displayed equal levels of IFN-γ and CD107a expression. Next, we explored which NK cell subset is responsible for the enhanced cytokine production of CAR MLNK cells. Three NK cell subsets, CD56^bright^ CD16^+^, CD56^bright^ CD16- and CD56^dim^ CD16^-^ NK cells, were recognized in CAR MLNK cells following tumor stimulation (**Figure 3C**). CD56^bright^ CD16^-^ NK cells, known as NK cell cytokine producer, expressed the highest levels of IFN-γ and CD107a, followed by CD56^dim^ CD16^-^ subset and CD56^bright^ CD16^+^ subset. CD56^bright^ CD16^-^ cell population expressed higher levels of IFN-γ in CAR MLNK cells compared with CAR NK cells when in response to antigen stimulation (**Figure 3D**, 30.7 ± 1.6% vs 26.5 ± 0.8%, n = 4). CD56^bright^ CD16^+^ subset in CAR MLNK cells also exhibited increased expression of IFN-γ (13.5 ± 2.0% vs 9.8 ± 1.8%) as well as CD107a (31.8 ± 1.9% vs 26.4 ± 1.1%), whereas their expression were comparable in CD56^dim^ CD16^-^ subset. These data indicated that CD56^bright^ CAR MLNK cells displayed enhanced degranulation and IFN-γ production in response to CD19^+^ tumor stimulation.

### CAR MLNK cells exhibited enhanced survival and cytotoxicity against CD19^+^ tumor cells *in vitro*

**Figure 4:**
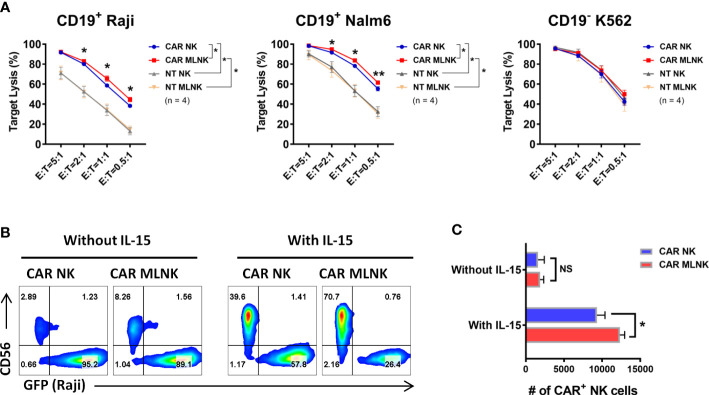
Cytotoxic capacity of CD19 CAR engineered memory-like NK cells. **(A)** Cytotoxicity of CAR NK cells, CAR MLNK cells, NT NK cells or NT MLNK cells against CD19^+^ Raji, CD19^+^ Nalm6 and CD19^-^ K562 expressing Firefly luciferase was determined by luminescence. % Target Lysis  = 100% × (RLU of untreated tumor cells – RLU of tumor cells cultured with NK cells)/RLU of untreated tumor cells (n = 4). **(B)** Representative flow cytometry plots showing the percentage of CAR MLNK cells or CAR NK cells after cocultured with CD19^+^ Raji expressing GFP for 3 days at E/T ratio of 1:4 in the presence of 10 ng/mL IL-15 or not. **(C)** Summary data of absolute numbers of remaining CAR MLNK cells or CAR NK cells after 3 days coculture (n = 4). *P < 0.05; **P <0.01; ns, no significance.

Next, the antigen-specific cytotoxic capacity of CD19 CAR MLNK cells was investigated. CD19^+^ Raji lymphoma cells, CD19^+^ Nalm6 lymphoblastic leukemia cells and CD19^-^ K562 myelogenous leukemia cells were used as targets. CD19-specific CAR MLNK cells showed stronger lytic activity of CD19^+^ Raji and Nalm6 cells but not CD19^-^ K562 cells, when compared with non-transduced MLNK cells (**Figure 4A**). CAR MLNK cells were then compared to conventional CAR NK cells for the lytic capacity of CD19^+^ targets. Increased lysis of CD19^+^ Raji and Nalm6 cells were observed in CAR MLNK cells, particularly at low effector/target (E/T) ratios when comparing to CAR NK cells, while CD19 ^-^ K562 killing were comparable. The lytic activity between non-transduced MLNK cells and non-tranduced NK cells was comparable regardless against CD19^+^ or CD19^-^ tumor cells. To assess the survival of CAR MLNK cells after exposure to CD19^+^ targets, CAR MLNK cells were cocultured with CD19^+^ Raji cells at 1:4 E:T ratio for 3 days in the presence of IL-15 or not (**Figures 4B, C**). Similar to CAR NK cells, poor survival of CAR MLNK cells was observed after 3 days coculture without IL-15. Addition of IL-15 resulted in a significant increased of both CAR NK cells and CAR MLNK cells. Notably, the percentage and absolute number of CAR MLNK cells were significantly higher than that of conventional CAR NK cells following cocultured with CD19^+^ tumor cells in the presence of IL-15 (**Figure 4C**). Taken together, CD19 CAR modified memory-like NK cells showed enhanced *in vitro* survival and antitumor effects compared to conventional CAR NK cells.

### CAR MLNK cells displayed superior *in vivo* persistence and anti-tumor efficacy compared with conventional CAR NK cells

**Figure 5:**
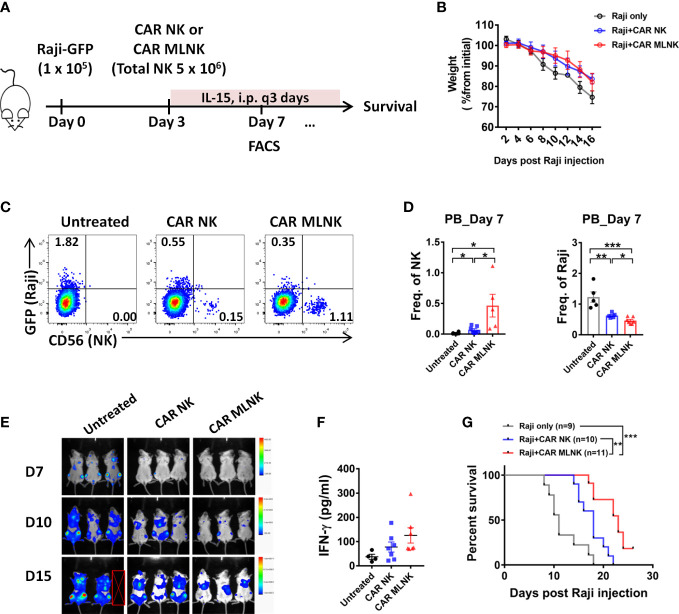
Antitumor efficacy of CD19 CAR MLNK cells *in vivo.***(A)** Schema of *in vivo* studies. Briefly, NSG mice received 1 × 10^5^ Raji cells IV followed by 5 × 10^6^ NK cell IV injection on day 3, containing 45% to 55% CAR positive NK cells or MLNK cells. **(B)** Body weight of mice were monitored every 2 days. **(C)** Representative FACS plots showing CAR NK cells, CAR MLNK cells and Raji cells in the peripheral blood on day 7 post Raji injection. **(D)** Summary data of human NK (CD56^+^ GFP^-^) cells and Raji (GFP^+^ CD56^-^) cells in the PB on day 7. **(E)** Tumor burden were monitored using IVIS, and representative data of the bioluminescence mice are shown. **(F)** The serum levels of IFN-γ on day 7 by ELISA were shown. **(G)** Kaplan-Meier survival curve of mice receiving tumor only (gray line, n=9), CAR NK cells (blue line, n=10), CAR MLNK cells (red line, n=11). Data was pooled from two independent experiments. *P < 0.05; **P < 0.01; ***P < 0.001; ns, no significance.

We next examined the anti-tumor activity of CAR MLNK cells *in vivo*. NSG mice were infused IV with GFP-expressing Raji cells and a single injection of CAR NK cells or CAR MLNK cells (**Figure 5A**). Previous studies demonstrated that MLNK cell survival in mouse xenograft models requires IL-15 or IL-2, similar to conventional NK cells (15, 22, 23). Therefore, low-dose of IL-15 was administered to support *in vivo* NK survival. Compared with NK cell-treated mice, untreated tumor-bearing mice showed a significant reduction of body weight from day 8 (about 10% weight loss) post tumor injection (**Figure 5B**), suggesting tumor progression in these mice. On day 7, higher frequency of NK cells in peripheral blood (PB) was found in CAR MLNK cell-treated mice compared with conventional CAR NK cell-treated mice, indicating better expansion of CAR MLNK cells *in vivo*. Fewer tumor cells were found in the PB of mouse received CAR NK or CAR MLNK cell therapy compared with untreated mouse as anticipated. Notably, frequency of Raji cells as well as the tumor burden assessed by IVIS in CAR MLNK-recipient mice was lower than that in CAR NK-treated mice (**Figures 5C–E**), suggesting that CAR MLNK cells exhibited stronger anti-tumor activity than CAR NK cells. We also investigated the levels of IFN-γ in the serum on day 3 after NK cell infusion and found IFN-γ was increased in the mice treating with CAR MLNK cells compared with CAR NK cells (**Figure 5F**). Finally, the enhanced *in vivo* persistence of CAR MLNK cells resulted in improved survival of treated mice compared with conventional CAR NK cells (**Figure 5G**). These data demonstrated that CAR MLNK cells provide enhanced tumor control *in vivo*.

## Discussion

In this study, we demonstrated that NK cells pre-activated with IL-12/-15/-18 prior to transduction of CD19 CAR resulted in a more activated, non-exhausted and memory-like phenotype associated with increased IFN-γ production, degranulation, and specific killing against CD19^+^ leukemia and lymphoma cells. Moreover, memory-like NK cells that expressed an CD19 CAR exhibited enhanced persistence *in vivo* and improved the survival of CD19^+^ tumor bearing mouse, compared with conventional NK cells expressing CD19 CAR.

Immunological memory is the ability of the immune system to respond more rapidly and effectively to pathogens that have been encountered previously, and provides long-lasting and enhanced antigen-specific immunity (24). Memory-like NK cell responses have been defined following exposure of NK cells to specific hapten (25), viral infection (26), or combined cytokine activation with IL-12, IL-15, and IL-18 (15). Cytokine induced memory-like NK cells have been used for treating patients with relapse or refractory AML (16, 17), and MLNK cells are able to persist for at least 2 months *in vivo* (27). These clinical observations as well as preclinical studies suggest that MLNK cells might be an attractive cellular platform for genetically modified adoptive cell therapy.

Low transduction efficiency in primary NK cells is currently one of the major challenges for NK cell-based immunotherapy approaches (28). A recent report showed that modification of memory-like NK cells by lentiviral transduction showed only 13% of transduction efficiency (23). Here, our results show that memory-like NK cells expanded with K562 feeder cells and transduced with CAR retroviral vectors, resulted in nearly 80% transduction efficiency in MLNK cells, and its expression remained stable after 10 days *ex vivo* expansion (70.03 ± 2.37%). Compared to lentiviral vectors, the efficiency of retroviral transduction in NK cells is higher, and thus retrovirus has been widely used to generate CAR-NK cells in recent preclinical and clinical studies (5, 29–31). Stimulation with NK-sensitive K562 cells is known to augment NK cell expansion, and engineered K562 cells have been successfully used to generate large numbers of NK cells or CAR-NK cells for phase 1 and 2 clinical trials (5, 29, 32). We achieved efficient expansion and high transduction efficiency of CAR MLNK cells that were stimulated by irradiated K562 cells co-expressing CD48, CD137L and mbIL-21.

Unlike T cell memory populations, the phenotype of memory-like NK cells has not been fully elucidated. Romee et al. reported that human MLNK cells differentiated *in vitro* resulted in increased expression of NKG2A, CD94, NKp46 and CD25 (16). Here, we revealed a distinct phenotype profile of CAR MLNK cells, as evidenced by increased CD94 expression but reduced NKp30 and KIR expression, and no significant increased expression of NKp46, NKG2A and CD25 was observed in CAR MLNK cells. This discrepancy may be due to the introduction of CAR gene into MLNK cells in our study. Ewen et al. have shown that activation of NK cells with IL-12/-15/-18 reduced self-inhibition by KIR through downregulation of inhibitory receptors KIR2DL1, KIR2DL2/L3 and KIR3DL1 (33). Indeed, we confirmed lower expression of KIR2DL1 on CAR MLNK cells, which might unleash them from KIR/HLA-I inhibition and thus enhance killing of CD19^+^ tumor cells.

In human peripheral blood, NK cells are primarily divided into two subtypes: CD56^dim^ CD16^+^ and CD56^bright^ CD16^-^ cells. Here, four NK cell subtypes can be recognized during the generation and expansion of CAR MLNK cells, including CD56^bright^ CD16^+^ cells, CD56^bright^ CD16^-^ cells, CD56^dim^ CD16^+^ cells and CD56^dim^ CD16^-^ cells. CD56^bright^ CD16^+^ NK cells are less than 1% in PBMC but constitute the major population of CAR MLNK cells (86.1%). Previous study has demonstrated that CD56^bright^ CD16^+^ NK cells have potent cytotoxicities against not only MHC class I^-^ but also MHC class I^+^ tumors (34). The evolution of NK cell subsets in CAR MLNK cells was similar to conventional CAR NK cells, but CD56^bright^ CD16^-^ population of CAR MLNK cells expressed a significant higher levels of IFN-γ than that of conventional CAR NK cells, resulting in enhanced selectively killing of CD19^+^ tumor cells including NK-resistant lymphoma cells, as compared with conventional CAR NK cells.

The expansion and/or persistence of adoptive transferred cells are the major predictors of the therapeutic efficacy of adoptive cellular therapy (5, 35). Previous studies have demonstrated that MLNK cells exhibited superior *in vivo* expansion and/or persistence compared to conventional NK cells (15, 22), but comparisons on CAR MLNK cells and conventional CAR NK cells *in vivo* remains unclear. In this study, our results show that CAR MLNK cells exhibited enhanced expansion and persistence in a Burkitt’s lymphoma mouse model, resulting in improved survival of lymphoma-bearing mice compared with conventional CAR NK cells. Upon further analysis of serum cytokine levels, IFN-γ was elevated while IL-6 was undetectable (data not shown) in our immunodeficient mouse model. Whether CAR MLNK cells could induce or increase the incidence of CRS remains unknown, thus, further studies with humanized mouse models are warranted to explore the potential toxicity of CAR MLNK cell therapy.

In conclusion, we demonstrated that CD19 CAR engineered memory-like NK cells exhibited enhanced antitumor efficacy against CD19^+^ leukemia cells and lymphoma cells both *in vitro* and *in vivo*, as well as increased persistence in a mouse xenograft model of lymphoma compared with the conventional CAR NK cells, providing an attractive approach for treating patient with relapse or refractory B cell malignancies. Memory-like NK cells expressing tumor-specific CARs can be used to target a wide range of antigens and indications including solid tumors. Thus, further investigations are warranted to measure the clinical efficacy of CAR memory-like NK cells in the treatment of hematological and solid tumors.

## Data availability statement

The raw data supporting the conclusions of this article will be made available by the authors, without undue reservation.

## Ethics statement

The studies involving human participants were reviewed and approved by the Ethics Committee of Nanfang Hospital, Southern Medical University. The patients/participants provided their written informed consent to participate in this study. The animal study was reviewed and approved by the Ethics Committee of the University of Southern Medical University.

## Author contributions

QL and CL took part in study conception and design. BH and QM performed the experiments, analyzed the data, and wrote the manuscript. YP, SD and RX cloned the retroviral vector and helped with the animal experiment. YH, NX, LX, HZ and XL provided helpful suggestions. All authors contributed to the article and approved the submitted version.
