# Supplementary material for: Cytokines induced memory-like NK cells engineered to express CD19 CAR exhibit enhanced responses against B cell malignancies
Source: Front Immunol. 2023 May 3;14:1130442. doi: 10.3389/fimmu.2023.1130442 (PMC10191231; doi:10.3389/fimmu.2023.1130442)
Supplement: Supplementary file 1 [file Presentation_1.pptx]

## Slide 1
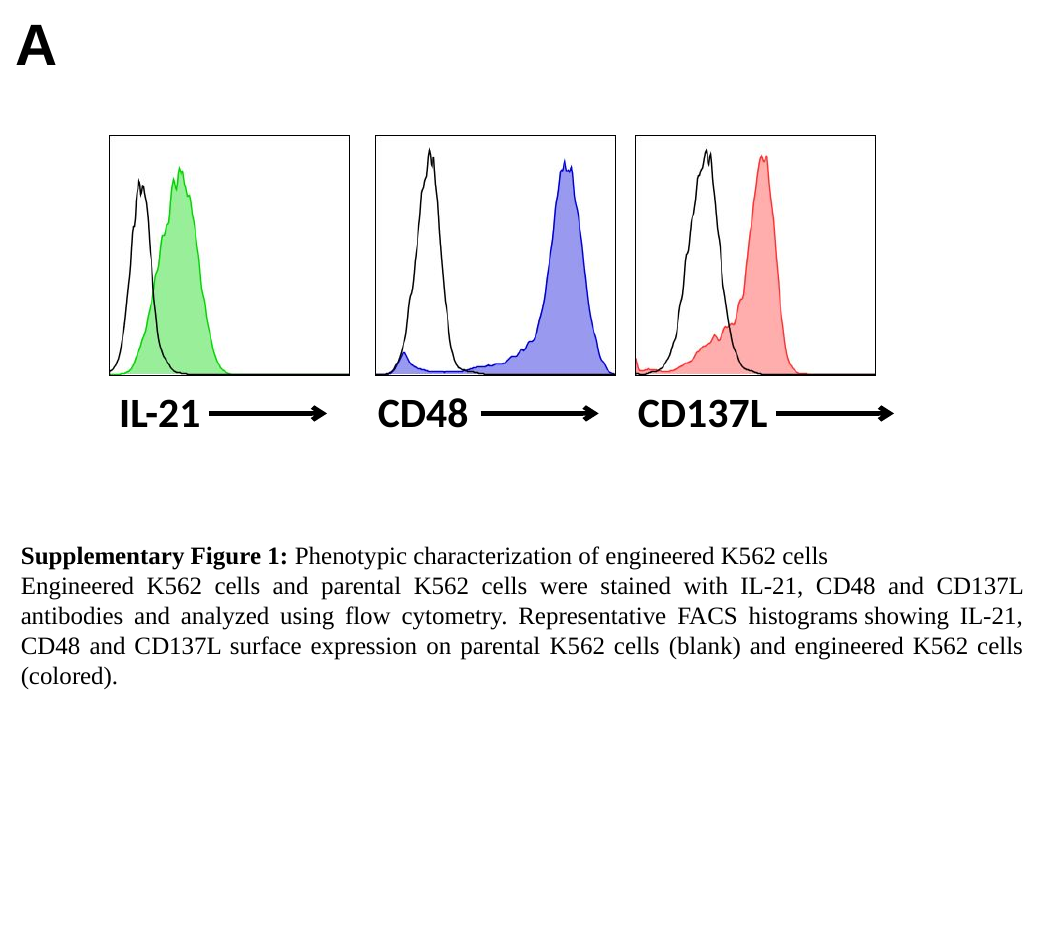

A
IL-21
CD48
CD137L
Supplementary Figure 1: Phenotypic characterization of engineered K562 cells
Engineered K562 cells and parental K562 cells were stained with IL-21, CD48 and CD137L antibodies and analyzed using flow cytometry. Representative FACS histograms showing IL-21, CD48 and CD137L surface expression on parental K562 cells (blank) and engineered K562 cells (colored).

## Slide 2
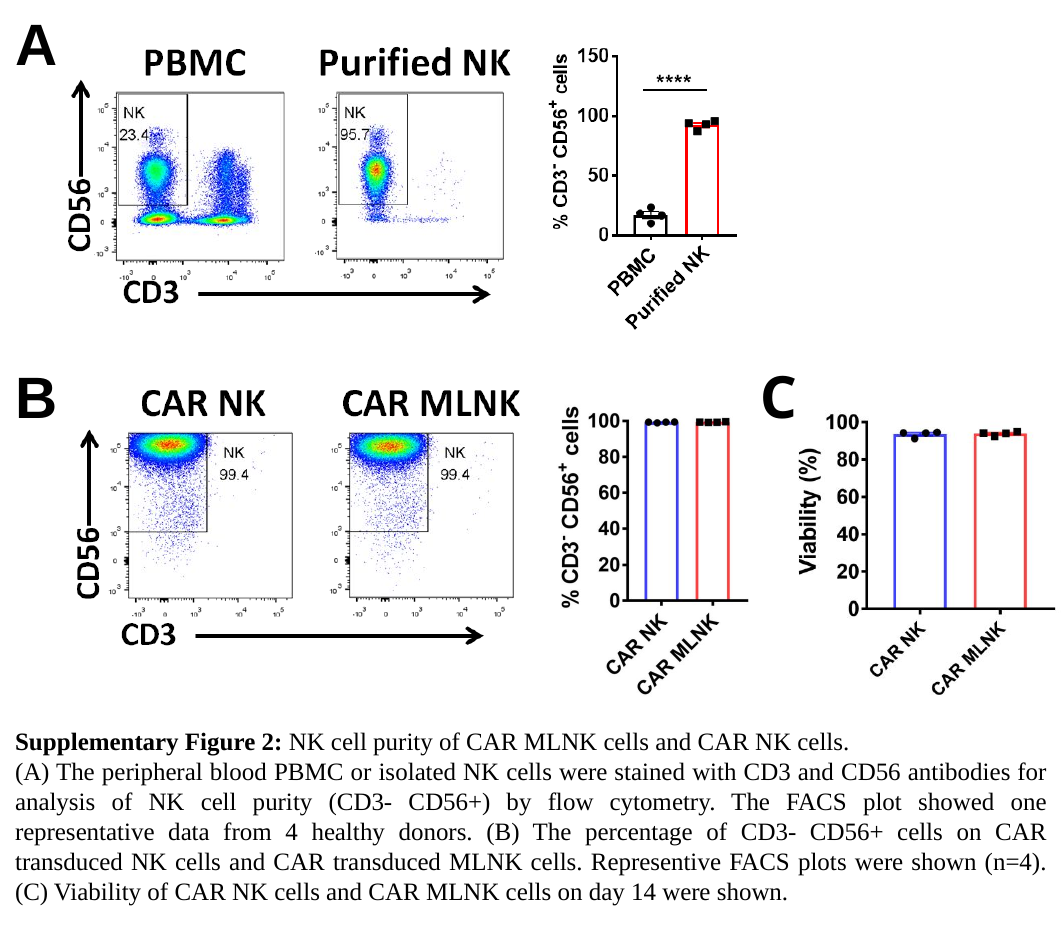

A
B
C
Supplementary Figure 2: NK cell purity of CAR MLNK cells and CAR NK cells.
(A) The peripheral blood PBMC or isolated NK cells were stained with CD3 and CD56 antibodies for analysis of NK cell purity (CD3- CD56+) by flow cytometry. The FACS plot showed one representative data from 4 healthy donors. (B) The percentage of CD3- CD56+ cells on CAR transduced NK cells and CAR transduced MLNK cells. Representive FACS plots were shown (n=4). (C) Viability of CAR NK cells and CAR MLNK cells on day 14 were shown.

## Slide 3
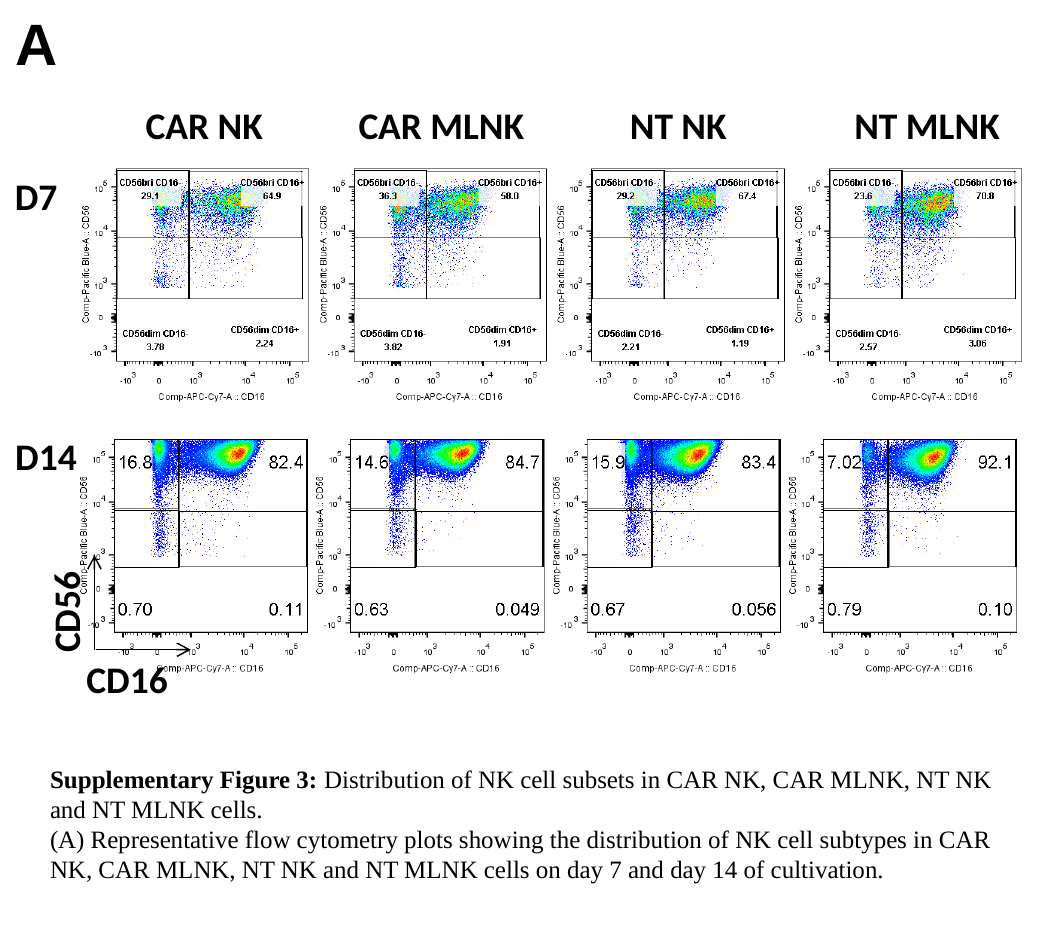

A
CAR NK
CAR MLNK
NT NK
NT MLNK
D7
D14
CD56
CD16
Supplementary Figure 3: Distribution of NK cell subsets in CAR NK, CAR MLNK, NT NK and NT MLNK cells.
(A) Representative flow cytometry plots showing the distribution of NK cell subtypes in CAR NK, CAR MLNK, NT NK and NT MLNK cells on day 7 and day 14 of cultivation.
